# The Use of Phage Display and Yeast Based Expression System for the Development of a Von Willebrand Factor Propeptide Assay: Development of a Von Willebrand Factor Propeptide Assay

**DOI:** 10.1155/2018/6232091

**Published:** 2018-05-24

**Authors:** S. M. Meiring, B. D. P. Setlai, C. Theron, R. Bragg

**Affiliations:** ^1^National Health Laboratory Services, Universitas Hospital, South Africa; ^2^Department of Haematology and Cell Biology, University of the Free State, Bloemfontein 9301, South Africa; ^3^Department of Microbial, Biochemical and Food Biotechnology, University of the Free State, Bloemfontein 9301, South Africa

## Abstract

**Background:**

The diagnosis of von Willebrand disease is complex due to the heterogeneity of the disease. About eighty percent of von Willebrand disease patients are diagnosed with a quantitative defect of von Willebrand factor (VWF) where fifty percent is due to an increased clearance of von Willebrand factor. These patients do not respond well to the treatment of choice, Desmopressin (DDAVP) due to decreased efficacy. The ratio between the VWF propeptide and the mature VWF antigen is used to diagnose these patients. Commercial VWF propeptide assays are too expensive for use in developing countries. In this study, we developed a cost-effective ELISA assay.

**Methods:**

We first displayed VWF propeptide on yeast. Antibody fragments were selected against the displayed VWF propeptide by using phage display technology. The antibodies were used to develop a cost-effective VWF propeptide assay and compared to a commercial VWF propeptide assay.

**Results:**

Two of these antibody fragments bound specific to the VWF propeptide and not to the yeast used for the expression of the propeptides. These purified antibody fragments were able to detect VWF propeptide in normal plasma.

**Conclusion:**

Our assay performed well when compared to a commercial kit. It also showed a higher binding affinity for VWF propeptide in plasma at especially lower plasma concentrations.

## 1. Background

Von Willebrand disease (VWD) is the most common bleeding disorder in the world with a prevalence of one percent in the general population [1]. It is classified into three types. Types 1 and 3 are quantitative defects of von Willebrand factor (VWF) and Type 2 is a qualitative defect VWF [1]. The diagnosis of VWD is complex due to the heterogeneity of the disease. Various mutations of the VWF gene result in a variety of phenotypes that makes the diagnoses of these patients very challenging [1]. It is therefore not surprising that patients with VWD are largely under-diagnosed or misdiagnosed. About 80% of VWD patients are diagnosed with type 1 VWD and about fifty percent of these patients present with an increased clearance rate of VWF [1]. Patients with increased clearance of VWF need to receive different treatment strategies and the correct diagnosis is crucial, since DDAVP, the treatment of choice for type 1 VWD patients, would not be effective at all in these patients. An increased ratio between von the Willebrand factor propeptide (VWFpp) and the mature VWF antigen can be used to diagnose these patients. This can be interpreted as a shortened half-life of plasma VWF and therefore increased clearance of VWF [1].

Furthermore, the level of VWFpp in the circulation can also be used as a marker of VWF synthesis. Studies showed that VWFpp levels are more reliable than mature VWF levels in the assessment of endothelial cell activation and endothelial damage, since the plasma VWFpp concentration is not influenced by blood groups or factors such as adhesive properties and catabolism [2]. Increased plasma VWFpp concentrations have been described in vasculopathies such as hypertension and diabetes, systemic sclerosis, myocardial infarction, and cardiovascular mortality [2, 3].

The current commercially available assays that are used to test the levels of VWFpp in plasma are expensive and the price that medical aids pay does not cover the costs of the tests. With this research, we developed a cost-effective VWFpp diagnostic laboratory assay using antibodies produced by phage display technology. Phage display is an* in vitro* method that allows rapid production of antibodies during cycles of biopanning and propagation without the use of animal models [4–6].

## 2. Methods

### 2.1. Display of VWF Propeptide on Yeast

Since a commercial preparation of the VWFpp is not available, the VWFpp (P04275, amino acids 23-763 of VWF) were displayed on yeast according to the method described by Lin-Cereghino et al. (2005) [7]. In short, the VWF propeptide-encoding sequence was transferred from the pMK-RQ plasmid to pINA1317-CWP110 using the* Sfi*I and* Hind*III restriction sites. Recombinant expression vectors were used to transform the* Y. lipolytica *strain P01h. All plasmids and related reagents were purchased from TaKaRa, Japan. A negative control strain was constructed by transformation of* Y. lipolytica *strain P01h with the original pINA1317-CWP110 vector. Transformants were randomly selected from yeast nitrogen base selective plates [yeast nitrogen base without ammonium sulphate and amino acids: 0.17%, ammonium chloride: 0.4%, glucose: 1%, casamino acids: 0.2%, agar: 2%, and pH: 6.5]. Chromosomal integration in transformants was confirmed by PCR using genomic DNA as template.* Y. lipolytica *transformants were cultivated in yeast extract peptone dextrose broth [yeast extract: 1%, peptone: 2%, and glucose: 2%] on a rotary shaker at 30°C. After 48h cultivation, cells were harvested by centrifugation at 5000* g *for 5 min. Cell pellets were washed with phosphate buffered saline (PBS) and resuspended to a final concentration of 50 g/L wet cell weight prior to assay. A control yeast with no VWFpp displayed on it was also prepared.

To detect the VWFpp on the yeast cells, VWFpp displaying yeast cells and control yeast cells were coated onto a 96-well plate (Nunc, Thermo Scientific, USA) at decreasing concentrations ranging from 27.5 g/L to 0 g/L (only PBS) wet cell weight and fixed onto the plate by adding ice cold methanol (100%) to each well and dried again overnight at 37°C. After blocking with PBS/0.1% Tween-20 with 1% Bovine Serum Albumin for 2hrs at 37°C, the plate was washed three times with PBS/0.1% Tween-20. The bound VWFpp was detected with a commercial anti-VWFpp horseradish peroxidase-conjugated antibody (CLB-Pro 14.3, 1:1000 dilution in PBS/Tween/BSA, Biocom Africa, SA) at 37°C for 2hrs. The reaction was developed with* o*-phenylenediamine dihydrochloride (OPD) substrate (10ml 0.1M Citric acid, 10 ml 0.1M Na_2_HPO_4_, 200 *μ*l OPD (1mg/ml), and 8*μ*l H_2_O_2_) and stopped with 30*μ*l of 4M sulphuric acid after 15min. Absorbance was measured at 450 – 650nm using the SynergyHT ELISA reader (Biotek, USA).

### 2.2. Selection of Antibody Fragments

Two phage display libraries (Tomlinson I & J) from the Medical Research Council Centre for Protein Engineering, Cambridge in London, UK, were amplified. The I library consists of diversified side chains on 18 residues while the J library has NNK side chains on these residues (N=GACT and K=GT). Antibody selection was done as previously described [8]. In short, Nunc-Immuno™ tubes (Thermo Scientific, USA) were coated with VWFpp displaying yeast cells (110 g/l in PBS) and rotated overnight at 4°C. After washing (3 times) and blocking with 2% skimmed milk powder in PBS at room temperature for 2 hours, 10^12^ phages of each library (I and J) were added to the tubes and rotated at room temperature 2 hours. After 10 times washing, the unbound phages were discarded and the bound phages were eluted with 500 *μ*l trypsin-PBS (10mg/ml trypsin, 50mM Tris-HCl, 1mM CaCl_2_ in PBS, and pH7.4) for 10min at room temperature. TG1* E-coli *cells (1.75ml) were infected with the eluted phages for 30 minutes at 37°C in a water bath until OD_600_ reach 0.4-0.6. The infected TG1* E-coli* cells were then centrifuged at 11600g in a microcentrifuge for 5min; the pellet suspended in 50*μ*l 2TY medium (16g bacto-tryptone, 10g yeast extract, and 5g NaCl in 1 liter distilled water) and grown on trypsin-yeast plates (15g agar, 10g bacto-tryptone, 8g NaCl, and 5g yeast extract in 1 liter distilled water) containing 100 *μ*g/ml ampicillin and 1% glucose overnight at 37°C. Cells were loosened from the plates with 2ml 2TY medium containing 15% glycerol and a small volume (50*μ*l) was grown in 50ml 2TY containing 100 *μ*g/ml ampicillin and 1% glucose at 37°C until OD_600_ of 0.4-0.6 was reached. The culture was then infected with 5 × 10^10^ helper phages, incubated at 37°C in a water bath for 30 min, centrifuged at 3000g for 10min and resuspended in 50ml 2TY medium containing 100 *μ*g/ml ampicillin, 50*μ*g/ml kanamycin, and 0.1% glucose, and amplified overnight shaking at 30°C. After centrifugation at 3300g for 15min, poly-ethylene glycol was added to 40ml supernatant and mixed well and the phages were precipitated on ice for 2hrs. The phage mixture was centrifuged again at 3300g for 30min at room temperature, resuspended in PBS, and centrifuged shortly to remove access bacterial cells. The precipitated phages were used for the next round of selection. Three selection rounds were performed.

Ninety-six single colonies from the third selection round of the I and J libraries, respectively, were amplified in 96-well microculture plates. The individual colonies were grown overnight shaking (250 rpm) at 37°C in 100*μ*l 2TY media containing 100 *μ*g/ml ampicillin and 1% glucose. Two *μ*l of the above cultures was inoculated into each well of the plates containing 200 *μ*l 2TY with 100 *μ*g/ml ampicillin and 1% glucose and grown shaking at 37°C until an OD_600_ of 0.4-0.6 was reached. After infection with 1 × 10^9^ helper phages for 30 min, the cultures were pelleted and resuspended in 2TY medium containing 100 *μ*g/ml ampicillin and 50 *μ*g/ml kanamycin and grown overnight at 30°C. The cultures were then centrifuged at 1,800g for 10 min and the supernatant from each of the single colonies was tested for binding to the VWFpp.

Two ELISA plates (one of each library) were coated with the VWFpp displaying yeast and another two plates with the control yeast. Fifty *μ*l of the supernatant of each of the different monoclonal phage colonies was added to both plates and incubated for 2 hours at 37°C. The plates were then washed and 100*μ*l of a 1:5000 dilution of HRP-anti-M13 antibody (Amersham, South Africa) in PBS/2% skimmed milk was added and incubated for 1 hour at 37°C. After washing again, the reaction was developed as mentioned in previous section. The six colonies with the highest binding affinity (OD_490-630nm_ > 0.5) were upscaled and tested for concentration depended binding to VWFpp. The following phage concentrations were used: 5 × 10^10^ phages/ml, 2.5 × 10^10^, 1.25 × 10^10^, 6.25 × 10^9^, 3.125 × 10^9^ and a blank, etc. The two colonies with the strongest and most specific binding to the VWFpp on the displaying yeast were used to produced soluble antibody fragments.

### 2.3. Production of Soluble Antibody Fragments

Phages from each of the two strongest binders were used to infect exponentially growing HB2151 bacteria (OD_600_ of 0.4-0.6). In order to obtain soluble antibody fragments, isopropyl-*β*-D-thiogalactoside (IPTG) (Thermo Scientific, USA) was added to a final concentration of 1mM to the cultures and grown overnight at 30°C. The overnight cultures with IPTG were centrifuged at 1,800 g for 10min and the scFv in the supernatant was concentrated with the minimate™ TFF filtration system (Thermo Scientific, USA).

### 2.4. Protein Purification

A Protein A IgG purification kit (Thermo Scientific, USA) was used according to the instructions of the manufacturers. Elution fractions with the highest absorbance values were pooled together and dialysed in PBS overnight using a Slide-A-Lyzer Dialysis cassette (Thermo Scientific, USA) with 10 000kD cut-off and stored at 4°C for further use. The two purified antibody fragments were then characterised in a VWFpp assay and compared to commercial anti-VWFpp antibodies.

### 2.5. Assay Development

A sandwich ELISA was performed to show that the antibody fragments are able to identify the VWFpp in normal human plasma (WHO 6^th^ International standard for FVIII and VWF in plasma). One antibody fragment (A9) was used to coat the plate and the other one (G7) was conjugated with HRP (EZ-Link activated peroxidase antibody labelling kit, Thermo Fisher Scientific, USA) and used as the detection antibody. We also used our scFv as coating antibodies with commercially available antibody as the detection antibody and* vice versa*. Furthermore, the commercially available antibodies were also used alone for comparison. The outline of these assays was as follows:

1. Coating antibody: CLB-Pro 35, Detection antibody: CLB-Pro 14.3
2. Coating antibody: CLB-Pro 35, Detection antibody: A9
3. Coating antibody: A9, Detection antibody: CLB-Pro 14.3
4. Coating antibody: G7, Detection antibody: CLB-Pro 14.3
5. Coating antibody: A9, Detection antibody: G7.

Protein A-HRP was used as previously described for detection in the second scenario. The assay conditions were again similar to that of the polyclonal ELISA. This assay was repeated twice. As validation of our assay, we compared the standard curves of using both commercial antibodies and of using our two scFv. The sensitivity and interrun accuracy of the assay were also determined from the standard curve.

## 3. Results

**Figure 1:**
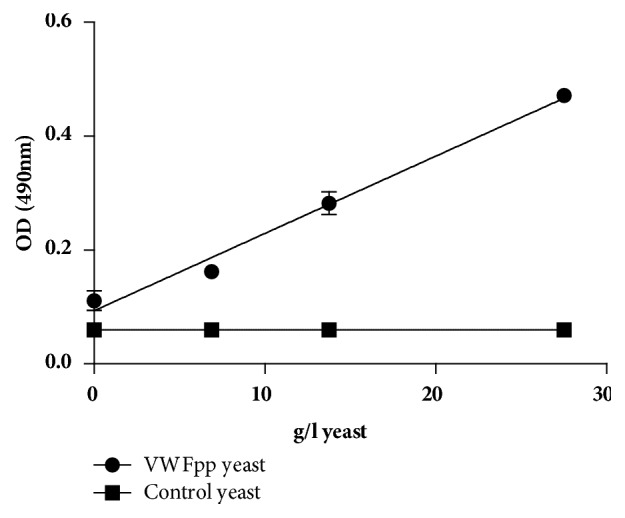
Existence of the displayed VWFpp. The VWFpp displayed yeast is indicated by dots and the control yeast indicated by squares. The binding is expressed as average ± 1 standard deviation (SD) where *n* = 2.

The displayed propeptide onto the yeast is confirmed in Figure 1. The displayed propeptide binds to the commercial antibody (CBL-Pro 35) that is directed against the VWFpp. The control yeast however did not bind to the anti-VWFpp antibody.

**Figure 2:**
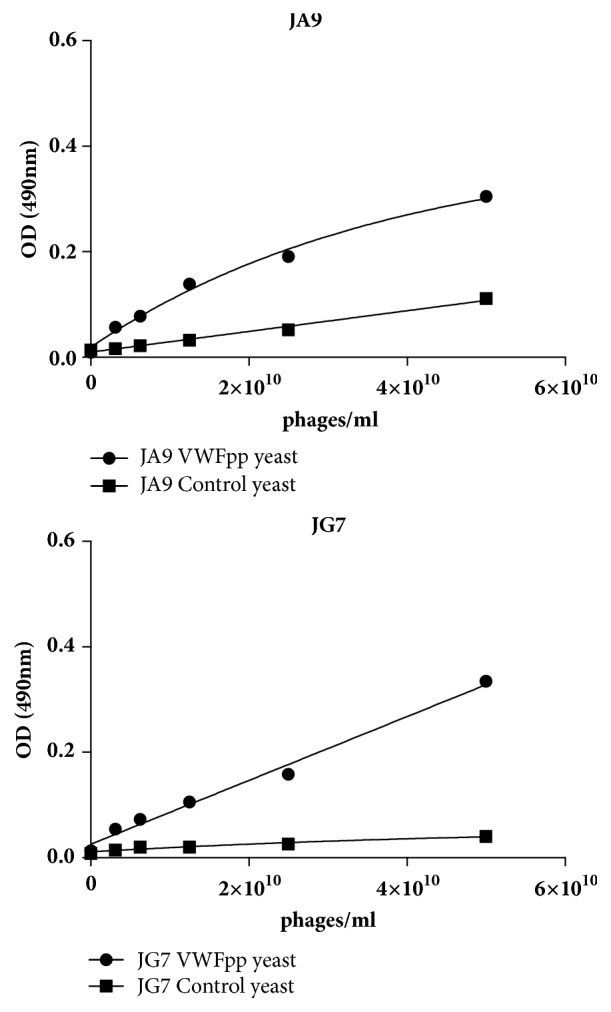
Concentration dependent binding of single colonies that showed specific binding affinity for VWFpp displayed on yeast. Phage binding to the VWFpp yeast is indicated by dots and to the control yeast by squares. The binding is expressed as average ± 1 standard deviation (SD) where *n* = 2.

From the third selection round, three colonies from the I library and 6 from the J library were identified as strong binders to the VWF displayed yeast and not the control yeast, but only two of them bound specific to the VWFpp yeast and not the control yeast. The results of the concentration dependent ELISA are shown in Figure 2. The two monoclonal phages, JA9 and JG7, showed the most specific affinity for VWFpp.

Soluble antibody fragments grown from these two colonies bound concentration dependently to the VWFpp displayed on the yeast. Neither of them bound to the yeast itself.

These two colonies were then purified on a protein A columns. Both proteins appeared in the first 2 fractions during the elution process. The protein concentrations of these antibody fragments were calculated as 185 *μ*g/ml for JA9 and 191 *μ*g/ml for JG7 after dialysis with PBS.

Our VWFpp assay where standard plasma (6^th^ WHO VWF: FVIII standard, NIBSC, UK) was used and compared to a commercial kit with antibodies (CLB-Pro 35 & CLB-Pro 14.3, Biocom Africa, SA).  Figure 4 showed that our assay using both our antibody fragments compared well to that of the assay using commercial antibody fragments. Our assay detects VWFpp concentrations form 1.5625%, while the commercial assay only detects VWFpp from 6.25%. Furthermore, a total %CV of 16% between our duplicate runs were found. Thus this standard curve serves as proof that our two antibody fragments are suitable to be used in a VWFpp assay.

The differences in the protein sequences of our JG7 and JA9 antibody fragments are shown in Box 1. The light and heavy chains of the two antibody fragments do differ and thus are suitable to be used in a sandwich ELISA assay, since it is unlikely that they might bind to the same binding site on the VWFpp.

The cost of producing 1 mg of a monoclonal antibody amounts to approximately 45 US Dollar (4500 USD for 100 mg). However, it will be much more cost-effective to produce single chain variable fragments in large scale using metal-affinity columns (about 3000 USD for 100 mg) [9]. The cost of our assay amounts to approximately 60% of the cost of a commercial assay kit. This will allow our medical aids to support funding for patients with increased VWF clearance who needs the outcome assay for diagnostic purposes.

## 4. Discussion

The VWFpp assay has recently been included in the diagnostic setup of von Willebrand disease (VWD) [10]. The ratio of the propeptide to antigen (VWFpp/VWF:Ag) is used to determine the clearance rate of VWF from the circulation [11]. The VWFpp assay also measures endothelial cell activation during endothelial damage [12, 13].

Since the current commercially available assays that measure the levels of VWFpp in plasma are so expensive, we developed a cost-effective VWFpp diagnostic laboratory assay using antibodies produced by phage display technology. Phage display technology was used because it is a molecular diversity technology that allows the presentation of large amounts of various proteins on the surfaces of filamentous phages. It also allows rapid production of antibodies during cycles of biopanning and propagation without the use of animal models. Phage display libraries thus permit the selection of peptides and proteins, including antibodies, with high affinity and specificity for almost any target [14]. The use of phage display also allows the user to manipulate the protocol according to the requirements of the target antigen. As long as the target is immobilised to a support and the exposed solutions containing phage are immobilised to the target, the changes of the experiment succeeding are almost a hundred percent [6]. We used two single chain variable antibody fragment libraries (Tomlinson I and J libraries) from which we selected the VWFpp binding antibody fragments [15].

The advantage of using small antibody fragments is that they easily penetrate the cellular or tissue membranes without compromising their affinity and specificity. Antibodies fragments are easier and faster to produce. They can be easily purified with commonly used purification systems such as protein A [16, 17].

The VWFpp is not commercially available and therefore we needed to express the recombinant protein.* E.coli* protein expression systems are widely used but lack proper protein folding and posttranslational modifications [17]. Mammalian cells produce very low yields and are also very costly [18]. We made use of a yeast display system to display the VWFpp antigen (amino acids 23-763 of VWF). Yeast display is more likely to produce soluble functional proteins with the appropriate posttranslational modifications [19]. Furthermore, proteins displayed on the surface of the yeast can be purified easily as an active yeast particle by centrifugation. A yeast cell is also capable of displaying up to 50–2000 copies of the antigen on its surface [20].

The combination of phage display and yeast expression systems in protein expression provides the opportunity to produce antibodies without the need of soluble target protein [21]. The reason why we did not use yeast display for the selection process is because the library sizes are smaller than those of other display systems. The existence of the displayed VWFpp on the yeast was confirmed (Figure 1).

We used the Tomlinson I and J Phage display single chain variable fragment libraries since they have a diversity of over 100 million different antibody fragments (Tomlinson I & J protocol). Three selection rounds are mostly used in literature [22] so we also performed 3 rounds of selection. Ninety-six colonies of each library's selection were chosen from the third selection round. Only two colonies (JA9 and JG7) showed specific binding to the VWFpp and not to the yeast (see Figure 2).

**Figure 3:**
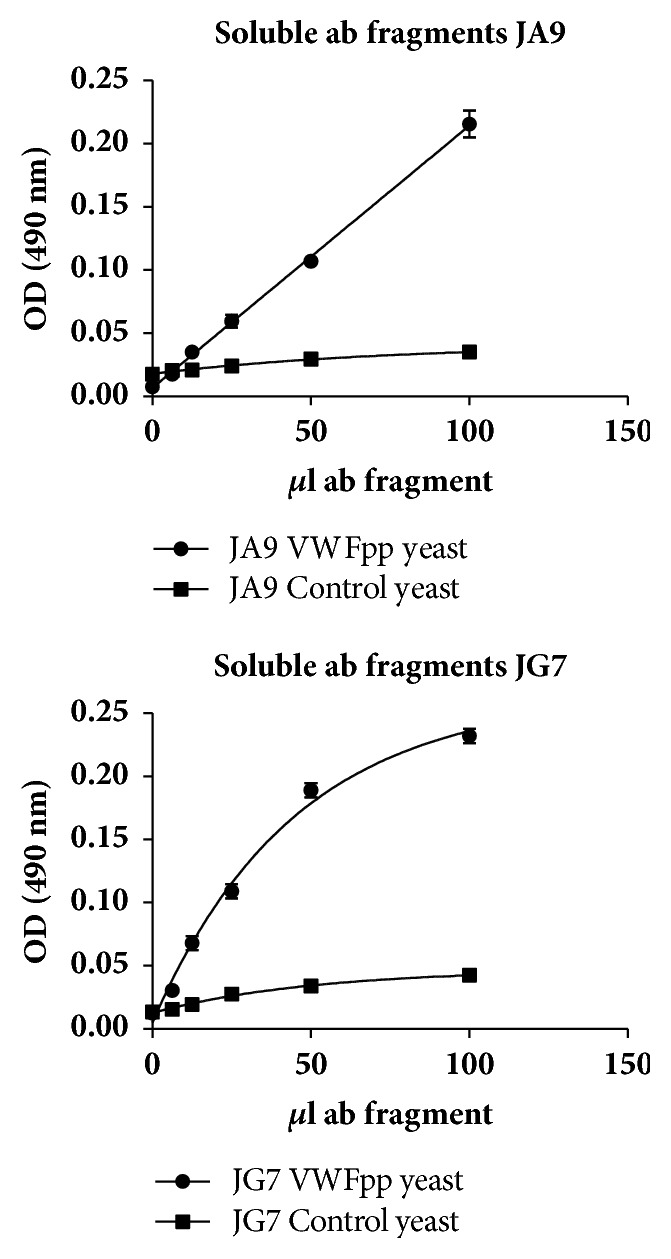
Concentration dependent binding curves of soluble single chain variable antibody fragments that bind specific to the VWFpp on the yeast. Binding to the VWFpp yeast is indicated by dots and to the control yeast by squares. The binding is expressed as average ± 1 standard deviation (SD) where *n* = 2.

**Figure 4:**
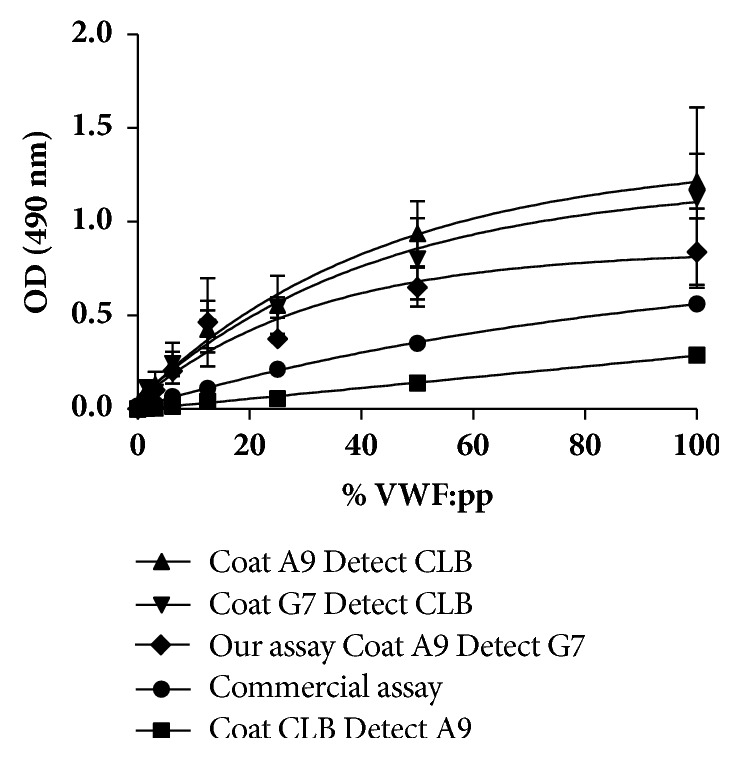
VWFpp ELISA assays with our 2 purified antibody fragments, commercially available VWFpp antibodies and combinations of both. Our assay (diamonds), commercial assay (dots), commercial coating antibody (ab) and JA9 detection ab (squares), JG7 coating ab and commercial detection ab (triangle upside-down) and JA9 coating ab, and commercial detection ab (triangle upright). The data are expressed as average ± 1 standard deviation (SD) where *n* = 2.

Some studies showed that phage colonies might lose their specificity after conversion into soluble antibody fragments [16, 22]. This however did not happen in our study. The antibody fragments of both colonies (JA9 and JG7) still bound concentration dependently to the displayed VWFpp and not to the control yeast (see Figure 3).

The availability of purification tags that can be used to purify antibodies generated with phage display makes the purification process easy and reliable [21]. The soluble scFv antibodies JA9 and JG7 were purified on protein A column and successfully eluted with 3-5ml elution buffer and aliquots pooled into single tube.

The amount of purified antibodies was just enough to perform a VWFpp assay where standard human plasma was used. The WHO 6^th^ International standard for FVIII and VWF in plasma was used, since it is the only standard with a given true value for the VWFpp. Although there is not a gold standard assay available to compare our assay with, we compared the standard curve of the VWFpp assay using our 2 antibody fragments to the standard curve of the VWFpp assay using two commercial antibodies to the VWFpp. Our assay shows higher binding affinity at especially lower plasma levels and might thus be more specific than the commercial one. Due to funding constraints and a lack of upscaling facilities, the amount of purified and concentrated antibody fragments was not enough to test patient samples and to do a full validation. However, the plasma that we used to set up a standard curve is of human origin and was pooled from between 20 and 40 human plasmas with an assigned value specific for the VWFpp.

The sensitivity of our assay was determined at 1.5625% VWF:pp in plasma, which is better than those of the commercial assay of 6.25%. The robustness of the assay was not determined in this study; however, it is known that the full-length VWF levels are influenced by cold storage at 4°C, but not by freezing at -80°C [23]. In this study, all plasma samples were stored at -80°C.

Specificity was however not determined in the final assay. We however showed that the soluble antibody fragments bind to the propeptide yeast and not to the control yeast; that indicated the specificity of the antibody fragments (Figure 3).

Commercial assays, however, show a high total %CV of 25% for the between-run accuracy [24]. We found a total %CV of 16% between our duplicate runs.

Since the amino acid sequence of the two antibody fragments differs significantly (Box 1), it might be used in a sandwich ELISA. The sequences were also compared to other scFv sequences in a database and more than 90% homology was found. It is known that all scFv from the I and J libraries are more than 90% homologous. No exact sequences to ours were found.

In conclusion, our antibody fragments can be used in a sandwich ELISA to determine the VWFpp levels in plasma. A follow-up study is however needed to fully validate the assay.

## Figures and Tables

**Box 1:**
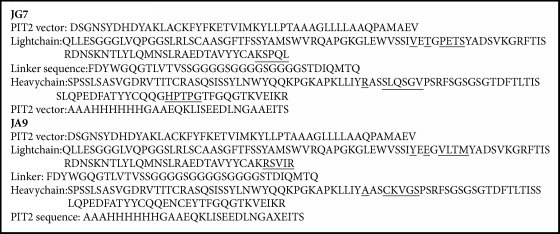
**Protein sequence of antibody fragments JA9 and JG7**. The differences between the two antibody fragments are underlined.
